# Multimodal Assessment of Recurrent MTBI across the Lifespan

**DOI:** 10.3390/jcm7050095

**Published:** 2018-05-01

**Authors:** Skadi Wilke, Kristin Prehn, Benedikt Taud, Jonathan List, Agnes Flöel

**Affiliations:** 1 Department of Neurology, Charité—Universitätsmedizin Berlin, 10117 Berlin, Germany; kristin.prehn@charite.de (K.P.); benedikt.taud@charite.de (B.T.); jonathan.list@posteo.de (J.L.); 2 Center for Stroke Research Berlin, Charité—Universitätsmedizin Berlin, 10117 Berlin, Germany; 3 NeuroCure Cluster of Excellence, Charité—Universitätsmedizin Berlin, 10117 Berlin, Germany; 4 Department of Neurology, University of Greifswald, 17475 Greifswald, Germany

**Keywords:** concussion, diffusion tensor imaging, voxel-based morphometry

## Abstract

Recurrent mild traumatic brain injuries (mTBI) and its neurological sequelae have been the focus of a large number of studies, indicating cognitive, structural, and functional brain alterations. However, studies often focused on single outcome measures in small cohorts of specific populations only. We conducted a multimodal evaluation of the impact of recurrent mTBI on a broad range of cognitive functions, regional brain volume, white matter integrity, and resting state functional connectivity (RSFC) in young and older adults in the chronic stage (>6 months after the last mTBI). Seventeen young participants with mTBI (age: 24.2 ± 2.8 (mean ± SD)) and 21 group-wise matched healthy controls (age: 25.8 ± 5.4 (mean ± SD)), as well as 17 older participants with mTBI (age: 62.7 ± 7.7 (mean ± SD)) and 16 group-wise matched healthy controls (age: 61.7 ± 5.9 (mean ± SD)) were evaluated. We found significant differences in the verbal fluency between young participants with mTBI and young healthy controls. Furthermore, differences in the regional volume of precuneus and medial orbitofrontal gyrus between participants with mTBI and controls for both age groups were seen. A significant age by group interaction for the right hippocampal volume was noted, indicating an accelerated hippocampal volume loss in older participants with mTBI. Other cognitive parameters, white matter integrity, and RSFC showed no significant differences. We confirmed some of the previously reported detrimental effects of recurrent mTBI, but also demonstrated inconspicuous findings for the majority of parameters.

## 1. Introduction

Recurrent mild traumatic brain injuries (mTBI) and its neurological sequelae have been the focus of a large number of studies over the last two decades. Moreover, the scientific findings were often intensively discussed in popular media, particularly with regard to well-known professional and amateur athletes [1,2].

However, scientific evidence in this area is far from unequivocal. In the acute phase (defined as 1 day to 1 week, post-injury), even non-symptomatic patients show lower performance in cognitive domains such as episodic memory, working memory, attention, and executive functioning [3]. However, most patients with mTBI experience full subjective recovery regarding clinical and cognitive symptoms within days or weeks.

In the chronic phase, defined as >6 months post-injury, a minority of patients reported persistent symptoms such as headaches, dizziness and fatigue (physical symptoms), cognitive impairments (for example, reduced concentration, memory dysfunction), and emotional distress (for example, depression, anxiety) [4]. In addition to these subjective complaints, objective evaluations revealed long-term cognitive sequelae and lower performance in memory tasks, attention, and executive functioning [5,6], similar to the acute phase after mTBI. Other reports, though, showed no group differences in cognitive tasks between patients in the chronic phase after mTBI and healthy controls [7]. Our own previous studies partly confirmed these reports showing only lower performance in verbal fluency tasks of young-to-middle-aged participants with mTBI in the chronic phase compared to group-wise matched healthy controls [8,9]. There is evidence that verbal fluency is not only a sensitive marker for cognitive deficits in the acute to subacute phase [10] and chronic phase [11] after mTBI but also for the early cognitive decline in patients with Alzheimer’s disease [12].

Of note, the age-related cognitive decline may be more pronounced due to the impact of recurrent mTBI [13] and the ensuing diminished cognitive reserve [14], with some studies even reporting a higher incidence of Alzheimer’s disease (AD) and dementia in older age [15,16].

With regard to neural correlates, magnetic resonance images (MRI) and computed tomography (CT) scans in the clinical setting reveal (per the definition of mTBI [17]) no overt brain lesions. However, mTBI in adolescence may cause long-term volume loss in several brain areas [18]. A general gray matter volume loss in the frontal [19] and temporal lobes [9] has been reported, but also parietal (for example, precuneus) [19] and subcortical regions such as the hippocampus [20,21], as well as white matter regions such as the corpus callosum (CC) [22], may be affected in the chronic phase. Also, a study of Monti and colleagues (2013) investigated the impact of mTBI in a young to middle-aged cohort in the chronic phase showing a significant age by group interaction within the right hippocampus, indicating smaller hippocampal volume due to the age and the history of mTBI [20]. A study in mice suggests a hippocampal vulnerability to repeated mTBI by showing increased cell damage in the hippocampi, hours after the traumatic events [23].

However, negative reports have been published, showing no significant volume loss in the chronic phase after mTBI [24,25,26,27].

Given that the traumatic event often causes microstructural changes such as diffuse axonal injuries (DAI) as a result of the stretching, straining, and shearing forces, the white and gray matter integrity, assessed by means of diffusion tensor imaging (DTI) including the assessment of fractional anisotropy (FA) and mean diffusivity (MD), several studies have evaluated the differences in these parameters between participants with mTBI and the controls. In the chronic phase, reports regarding these parameters are heterogeneous, showing alterations such as increased [28,29] and decreased FA in the corticospinal tract (CST) [30], increased [28] and decreased FA in the CC [26,31,32,33], decreased FA in superior longitudinal fasciculus (SLF), decreased FA in uncinate fasciculus (UF) [34,35], and decreased FA in the inferior longitudinal fasciculus (ILF) [35]. Other studies could not confirm these differences, though [36].

Alterations in neural networks due to the traumatic event have also been noted [37,38]. Shumskaya and colleagues (2012), for instance, demonstrated early (within 4 weeks post-injury) alterations in the resting state functional connectivity (RSFC), showing decreased activity within the motor-striatal and increased activity within the frontoparietal network, suggesting deficits in motor functioning (with regard to the information processing speed). Further alterations have been shown in the default mode network (DMN) [39,40]. A pilot study of Zhu and colleagues (2015) could also show alterations within the DMN in the acute phase after mTBI and over the recovery time of college football players, with concomitant cognitive deficits [41]. For the chronic phase, though, no consistent pattern for RCSF changes emerged so far, possibly due to the fact that alterations from the acute and sub-acute phase revert over time [40].

In sum, by focusing on single outcome measures in small (for example, 4 to 10 patients with mTBI [29,33]) and specific cohorts, the results of previous studies addressing sequelae in the chronic phase after recurrent mTBI demonstrated inconsistent results.

We, therefore, conducted an evaluation of the impact of recurrent mTBI on a broad range of cognitive functions, including verbal episodic memory, processing speed, verbal fluency, visuospatial skills and working memory, regional brain volume, white matter integrity and RSFC in a total of 34 young and older participants with mTBI in the chronic phase. Here, we hypothesized that participants with mTBI, as compared to their age-matched healthy controls, would score lower in cognitive tests, show reductions of gray matter volume in the frontal lobes [19], precuneus [19], putamen [42], and hippocampus [21], as well as reduced microstructural integrity, specifically in the CC [31], fornix [5], and UF [34], and alterations in RSFC [38,39,40,41].

## 2. Experimental Section

### 2.1. Participants

Seventy-seven participants were screened for the present study. After evaluation of the inclusion and exclusion criteria, 71 participants were included in the study. Fulfilment of the following inclusion criteria was required: (1) normal findings on neurological exam; (2) no intake of medication that influences the central nervous system; (3) no signs of severe cognitive deficits (Mini Mental State Examination (MMSE) ≥26) [43]; (4) no signs of relevant depression (Beck Depression Inventory (BDI) ≤12) [44]. Exclusion criteria comprised of the following: (1) history of moderate or severe TBI (all groups) or history of mild TBI (control group); (2) epilepsy; (3) history of alcohol and/or substance abuse; and (4) attentional deficits.

In total, 17 young participants with mTBI (24.2 years ± 2.8 (mean ± SD), 15.4 years of education, 2 women, 2 left-handed) and 21 group-wise matched healthy controls (25.8 years ± 5.4 (mean ± SD), 15.1 years of education, 2 women, 2 left-handed) were recruited from local sports clubs in Berlin and intranet advertisements on the website of the Charité-Universitätsmedizin Berlin (see Table 1).

Data from these young subjects have partially been reported in previous studies of our group [8,9]. Additionally, 17 older participants with mTBI (62.7 years ± 7.7 (mean ± SD), 15.7 years of education, 8 women) and 16 group-wise matched healthy controls (61.7 years ± 5.9 (mean ± SD), 15.5 years of education, 8 women) were recruited from local sports clubs and through intranet advertisements.

For diagnosis of mTBI, a standardized questionnaire was used. Diagnosis of mTBI required reporting of either confusion for less than 24 h and/or loss of consciousness for less than 30 min following head injury (American Academy of Neurology Practice, 1997). Participants with mTBI had to report a history of at least two mTBI (young participants with mTBI-number of TBIs: 3.1 ± 1.6, range 2–7; time since last TBI in months 13.3 ± 14.8 (mean ± SD), range 0–54; older participants with mTBI-number of TBIs: 2.5 ± 0.7, range 2–4; time since last TBI in months 447.8 ± 186.0, range 62–712) taking place at least 6 months prior to study. 

The study was approved by the local ethics committee of the Charité-Universitätsmedizin Berlin, Germany, and performed in accordance with the Declaration of Helsinki. All participants provided written informed consent before investigation and received financial compensation for their participation.

### 2.2. Study Outline

After study enrolment, all subjects underwent a medical examination and broad cognitive testing, including verbal episodic memory, processing speed, verbal fluency, visuospatial skills, and working memory. Subsequently, an MRI session was conducted in order to obtain anatomical and functional MRI data. A board-certified neuroradiology specialist evaluated the anatomical images of both groups. Visual inspection revealed no structural lesions.

### 2.3. Cognitive Testing

To detect deficits in domains previously reported as impaired after mTBI, a battery of standardized cognitive tests was conducted [9,20,45,46,47].

#### Verbal Episodic Memory

Verbal episodic memory was examined using the German version of the Auditory Verbal Learning Test (AVLT) [48] and its subscales to assess the verbal learning ability (sum of trials 1–5 of word list learning; AVLT, Sum 1–5) and retrieval from verbal memory, as tested by a delayed recall task 30 min later.

#### Processing Speed

The Trail Making Test (TMT) parts A and B [49] were conducted in order to assess the participants’ processing speed. For TMT-A, subjects were instructed to connect a set of 25 numbers as fast and as accurately as possible and for TMT-B to alternate between the connecting numbers and letters. The TMT score was calculated using the following formula: TMT score = (TMT-B − TMT-A)/TMT-A.

#### Verbal Fluency

The Regensburger Verbal Fluency Test (RWT) [50] was used to assess the general verbal fluency, using ‘S’ words and ‘G/R’ words (alternating order) for phonemic fluency and ‘food’ and ‘clothes/flowers’ (alternating order) for category fluency specifically. Besides an overall verbal fluency score using the means, the sum scores for the verbal fluency (‘S’ words; category ‘food’) and verbal flexibility (‘G/R’ words; categories ‘clothes/flowers’) were computed.

#### Visuospatial Skills

The Rey-Osterrieth Complex Figure Test (ROCF) [51] was used to examine the visuospatial skills and episodic memory in the visuospatial domain; first, participants were asked to copy a complicated figure freehand (recognition), and after a delay of 30 min, drawing it from memory (recall).

#### Working Memory

Working memory performance was assessed using the Digit Span forward and backward (taken from the Revised Wechsler Memory Scale) [52].

#### Statistical Analysis of Cognitive Data

The statistical analysis of cognitive data was performed using SPSS 22.0 (PASW, SPSS, IBM, Armonk, NY, USA). To enable direct the comparison of performance in different cognitive domains, the z-scores for each age group were calculated respectively (with a mean score of 0 and SD of 1 for the respective healthy controls). The following statistical analyses were conducted using z-scores instead of raw data. Before using parametric tests, the normal distribution was ascertained (histogram check, |skewness value| <1).

A 2 × 2 multivariate analysis of variance (MANOVA) with the factors group (participants with mTBI versus healthy controls) and age (young versus older) as between-subject factors was conducted in order to analyse the group-specific differences in the cognitive profiles. If the MANOVAs revealed a significant main effect of group, post hoc Student’s *t*-tests were used to evaluate group differences. No adjustments were made to the control for multiple comparisons. The level of significance for all analyses was set at α = 0.05 (two-tailed).

### 2.4. Magnetic Resonance Imaging (MRI)

#### 2.4.1. Data Acquisition

The MRI images were obtained on a 3 T Siemens TRIO MR system (Siemens AG, Erlangen, Germany) using a 12-channel head coil at the Berlin Center of Advanced Neuroimaging. Imaging comprised a high-resolution T1-weighted magnetization-prepared rapid acquisition with gradient echo (MPRAGE) anatomical scan (TR = 1900 ms, TE = 2.52 ms, 192 sagittal slices, voxel-size of 1 × 1 × 1 mm, flip angle = 9°) and a diffusion-weighted spin-echo echo-planar imaging (EPI) sequence (TR = 7500 ms, TE = 86 ms, 61 axial slices, voxel size of 2.3 × 2.3 × 2.3 mm; 64 directions with a b-value of 1000 s/mm^2^ and one b = 0). Functional scans were also obtained measuring blood oxygen level-dependent (BOLD) signal at rest using a T2*-weighted EPI sequence (TR = 2300 ms, TE = 30 ms, voxel size of 3 × 3 × 4 mm, flip angle = 90°). A total of 34 slices sampled for whole-brain coverage and across 150 time points (volumes) were collected. During the 6-min functional scan, subjects were instructed to keep their eyes closed and not to think of anything in particular. For diagnostic purposes, a fluid-attenuated inversion recovery sequence (FLAIR) was acquired.

#### 2.4.2. Voxel-Based Morphometry

Analysis of high-resolution anatomical images was conducted using the voxel-based morphometry (VBM) toolbox (VBM8; http://dbm.neuro.uni-jena.de/vbm/) in SPM8 (Wellcome Department of Cognitive Neurology, London; http://www.fil.ion.ucl.ac.uk/spm) implemented in MATLAB 7.9.0 (Mathworks Inc., Sherborn, MA, USA). Further information on the data pre-processing is described in the supplementary material.

Group comparisons in the local grey matter volume were calculated using a full factorial model comprising the factors group (participants with mTBI versus healthy controls) and age (young versus older). The absolute grey matter thresholds of 0.25 were used to prevent the edge effects located at the border regions of the tissue maps. Significant group differences had to survive a cluster-wise FWE threshold of *p* < 0.05 using an uncorrected cluster-defining threshold of *p* < 0.001 unless otherwise noted. Since we were interested in structural differences between groups in specific brain regions found to be affected in previous studies, we also applied a voxel-wise small-volume FWE correction (SVC) using a priori defined regions of interests (ROIs) for the left and right medial orbitofrontal cortex (OFC) [19], the left and right precuneus, [19] the left and right putamen [42], and the left and right hippocampus [21] obtained from the Automated Anatomical Labeling (AAL) atlas [53]. For explorative correlational analyses and illustration purposes, we extracted grey matter values within the left medial OFC, the right precuneus, and the right hippocampus (which were the only regions found to differ significantly in grey matter volume between the groups) using the MarsBaR toolbox (marsbar.sourceforge.net) based upon SPM8.

#### 2.4.3. Tract-Based Spatial Statistics and Probabilistic Tractography

Analysis of diffusion tensor images was done using the software packages FSL (http://fsl.fmrib.ox.ac.uk/fsl/fslwiki/) and FreeSurfer (http://surfer.nmr.mgh.harvard.edu/). The steps of pre-processing of the diffusion-weighted images included the correction for eddy currents and head motion and were followed by the extraction of non-brain tissue and fitting of a tensor model to obtain 3D maps of FA and MD [54].

Voxel-wise statistical analysis of the FA and MD data was carried out using the FSL package TBSS (Tract-Based Spatial Statistics) [55]. Find further details on data processing in the supplementary material.

In order to obtain the regions of interest of the fornix, left/right corticospinal tract (l/rCST), left/right uncinate fasciculus (l/rUF), and left/right cingulum (l/rC), probabilistic masks have been created using the Juelich histology [56] and the JHU white-matter atlas [57], respectively, as implemented in FSLView. After thresholding, the fornix mask at a probability of 0.5 and the l/rCST, l/rUF, l/rC, and CC mask at a probability of 0.2, the masks were binarized and transformed into individual diffusion spaces by using affine registration. The subject-specific masks were then used to obtain individual MD and FA values. MD and FA values were then entered into a 2 × 2 MANOVA with the factors group (participants with mTBI versus healthy controls) and age (young versus old) using SPSS 22.0 to analyse the group specific differences in MD and FA of the respective ROIs.

#### 2.4.4. Analysis of RSFC Data

Functional imaging data were pre-processed using the Data Processing Assistant for Resting State fMRI (DPARSF; http://www.restfmri.net/forum/DPARSF) [58], an automated pipeline for resting state fMRI data analysis based upon SPM8 (Statistical parametric mapping software; Wellcome Department of Cognitive Neurology, London, UK; http://www.fil.ion.ucl.ac.uk/spm) and implemented in MATLAB 7.9.0 (Mathworks Inc., Sherborn, MA, USA). Further details on data pre-processing are described in the supplementary material.

To analyse the RSFC of the left and right medial OFC, the left and right precuneus, the left and right putamen, and the left and right hippocampus [38,39,40,41], we used anatomical ROIs from the AAL atlas as seed regions. The ROIs for left and right brain regions were transferred into each subject’s native space. Then, for each participant and time point, the time course of the pre-processed BOLD signal averaged within each seed region was correlated with the BOLD signal in each voxel of the whole brain. The Pearson r correlation coefficient 3D maps were z transformed (Fisher’s z) resulting in spatial maps representing the voxel-wise strength of functional connectivity to the seed region (zFC maps). For further statistics at the group level, the functional images were spatially normalized using an MNI EPI template re-sampled into 3 × 3 × 3 mm isotropic voxels. Finally, the normalized functional data were smoothed with a 4 mm FWHM Gaussian kernel which is recommended for RSFC data [58]. The group differences in RSFC had to survive a cluster-wise FWE threshold of *p* < 0.05 using an uncorrected cluster-defining threshold of *p* < 0.001.

## 3. Results

### 3.1. Group Differences in Cognitive Data

The MANOVA revealed no significant overall main effect of the group (F (12,43) = 0.68, *p* = 0.77, Wilk’s Λ = 0.84, η^2^ = 0.16), but a significant main effect of age (F (12,43) = 7.82, *p* < 0.001, Wilk’s Λ = 0.31, η^2^ = 0.69). No significant age by group interaction (F (12,43) = 1.07, *p* = 0.41, Wilk’s Λ = 0.77, η^2^ = 0.23) was found. With regard to the single cognitive domain verbal fluency and the overall verbal fluency score, we found a significant main effect of group (F (1) = 4.64, *p* = 0.04, η^2^ = 0.08) and age (F (1) = 23.88, *p* < 0.01, η^2^ = 0.31). However, the age by group interaction (F (1) = 3.06, *p* = 0.09, η^2^ = 0.05) did not reach significance.

**Figure 1:**
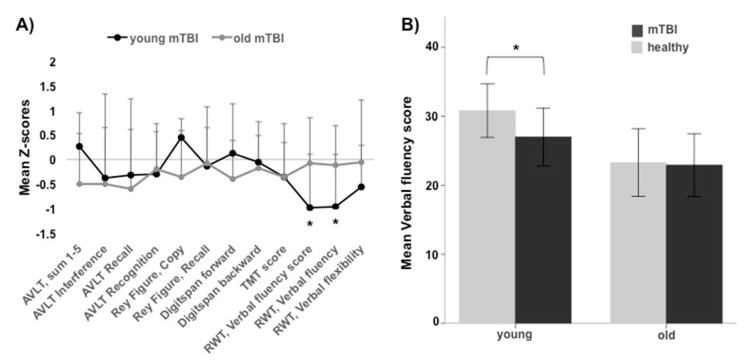
(**a**) The cognitive profiles of the young and older participants with recurrent mild traumatic brain injuries (mTBI), respectively. The mean *Z*-scores (+1 standard deviation) of the cognitive tests for participants with mTBI. (**b**) Mean Verbal fluency score as a function of group and age. * = *p* ≤ 0.05, error bars = ±1 standard deviation. mTBI = mild traumatic brain injury, AVLT = Auditory Verbal Learning Test; TMT-score = Trail Making Test score; RWT = Regensburg Verbal Fluency Test.

See Table 2 for the demographic data and the results of the post hoc Student’s *t*-tests regarding verbal fluency. Here, young participants with mTBI scored were significantly [uncorrected] lower (Figure 1) and both mTBI groups together (young + older) (Supplementary Figure 1) scored lower in the verbal fluency tasks ‘s words’ and ‘food’, though the last effect did not reach significance (see Table 2). See Supplementary Table 1 for the results of the post hoc Student’s *t*-tests regarding the other cognitive tests.

As indicated by the negative Z-scores, the mTBI groups performed lower in most of the cognitive tests (Figure 1).

### 3.2. Group Differences in Gray Matter Volume

**Figure 2:**
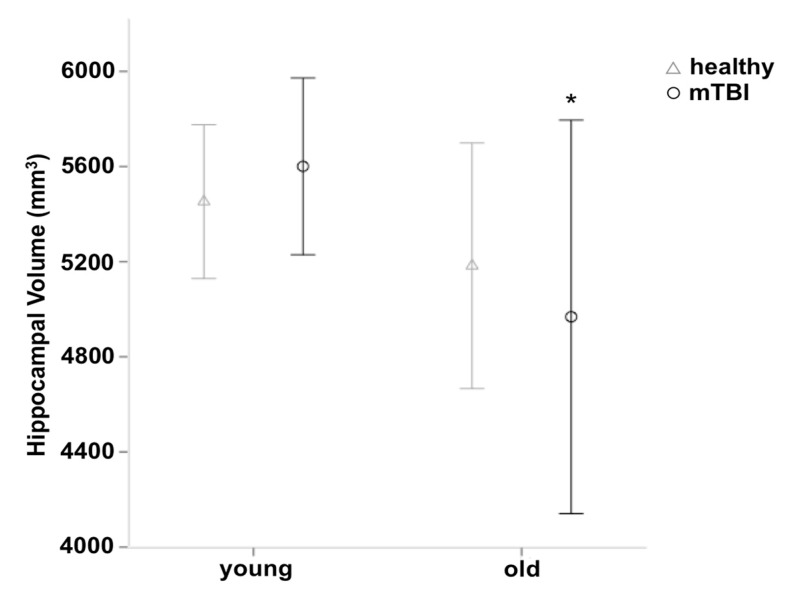
The hippocampal volume differences as a function of the group and age. mTBI = mild traumatic brain injury, mm^3^ = cubic millimeters, * = *p* ≤ 0.05, error bars = ±1 standard deviation.

The VBM analysis revealed no differences in grey matter volume between healthy controls and participants with mTBI at the whole brain level. Using voxel-wise SVC, we found grey matter volume reductions in the right precuneus (MNI-coordinate: 16/−64/40; *Z* = 3.59; 30 voxels) and the left medial OFC (MNI-coordinate: −14/64/−5; *Z* = 3.53; 21 voxels). As expected, this analysis also revealed large differences in the grey matter volume for young compared to older subjects including the middle frontal gyrus, the pre- and postcentral gyrus, the middle temporal gyrus, the hippocampus, the inferior parietal cortex, and the precuneus (see Supplementary Table 2). Using voxel-wise SVC, we also found an age by group interaction in the right hippocampus (MNI-coordinate: 38/−18/−23; *Z* = 3.16; 5 voxels), indicating a reduction of the grey matter volume in older compared to young participants with mTBI which was not present in older compared to young healthy controls (see Figure 2). Overall, the mTBI group (young + old) shows 0.79% less hippocampal volume, compared to the healthy control group. The present regions survived a cluster-wise FWE threshold of *p* < 0.05 using an uncorrected cluster-defining threshold of *p* < 0.01.

### 3.3. Group differences in DTI Measures

No significant differences between participants with mTBI and healthy controls were found using TBSS in FSL.

A MANOVA testing for group and age effects in FA in the different tracts revealed a significant main effect of age (F (4.60) = 9.80, *p* < 0.001, Wilk’s Λ = 0.61, η^2^ = 0.40) but not for group (F (4.60) = 0.33, *p* = 0.86, Wilk’s Λ = 0.97, η^2^ = 0.02). Furthermore, there was no significant age by group interaction (F (4.60) = 0.84, *p* = 0.50, Wilk’s Λ = 0.95, η^2^ = 0.05) found. For a detailed overview of the FA data, see Table 3.

Also, a MANOVA testing for the group and age effects in MD in the different tracts revealed no significant main effect of group (F (4.60) = 0.95, *p* = 0.44, Wilk’s Λ = 0.94, η^2^ = 0.06), but a significant main effect of age (F (4.60) = 10.04, *p* < 0.001, Wilk’s Λ = 0.60, η^2^ = 0.40). Furthermore, there was no significant age by group interaction (F (4.60) = 0.63, *p* = 0.65, Wilk’s Λ = 0.96, η^2^ = 0.04). For a detailed overview of the MD data, see Table 4.

### 3.4. Group Differences in RSFC

The analyses of RSFC of the right and left medial OFC, the right and left precuneus, the right and left putamen, as well as the right and left hippocampus revealed no differences between healthy controls and participants with mTBI. We only found changes in RSFC as a result of age (in RSFC of all brain regions of interest; see Supplementary Table 3). No significant age by group interactions or main effects of the group were found.

## 4. Discussion

In the present study, we found slight deteriorations in the cognitive abilities of young participants with recurrent mTBI but none for the older cohort. However, only the verbal fluency score reached significance, as already reported in previous studies of our group [8,9]. Further, a significant volume loss in the precuneus and medial OFC in young and older participants with recurrent mTBI was noted together with an age by group interaction in the right hippocampus, indicating a greater loss of volume in older participants with mTBI compared to young participants with mTBI.

### 4.1. Cognitive Differences between Participants with mTBI and Healthy Controls

Cognitive sequelae after recurrent mTBI have been well documented over the past years [11,13,59,60]. For example, studies reported participants with mTBI scoring lower in verbal fluency tasks in the acute and chronic phase after mTBI [11,60]. Mechanisms underlying a decline in cognitive abilities in the first years after recurrent mTBI are not yet resolved. It has been proposed that the structural and functional deficits such as the reduced cortical thickness in the right temporal lobe and left insula [9], decreased cellular integrity in the corpus callosum [18], cingulum [61], and fornix [5], as well as deficits in functional connectivity [38] due to the traumatic event, may account for the deterioration in cognition. Our data support the findings of lower performance in verbal fluency tasks, though, only apparent in the young cohort. One reason for this discrepancy might have been the large difference in time since the last mTBI between these two cohorts (young participants with recurrent mTBI–the time since the last mTBI: 13.3 ± 14.8 months (mean ± SD); old participants with recurrent mTBI–the time since the last mTBI: 427.8 ± 186.0 months (mean ± SD). This could be an indicator of a diminished impact of recurrent traumatic events over time. This assumption is supported by studies reporting differences in the acute and sub-acute phase after mTBI [60], but showing only little to no effects in the chronic phase [7].

### 4.2. Regional Volume Changes in Participants with mTBI

Acceleration–deceleration forces on the brain due to mTBI [62], supposedly affecting (amongst others) the frontal, parietal and occipital regions [63], may cause long-term regional and whole-brain volume loss [64]. Recent studies reported precuneal volume changes days and months after the traumatic event [65,66,67] and could show an association with memory deficits [66]. These results, however, were found after severe traumatic events rather than recurrent mTBI [65] and rather, in the acute and sub-acute instead of the chronic phase after mTBI [66,67]. Moreover, the volume loss within the OFC in the acute and sub-acute phase after mTBI has been reported and linked to the parallel increase in depression scores and decrease in cognitive performance [68]. However, several reports from the chronic phase could not confirm the volume changes after mTBI [24,25,26,27] indicating that the impact of the recurrent mTBI from the acute and sub-acute phase might diminish over time.

In the present study, small but significant volume differences were noted within the right precuneus and left medial OFC between participants with recurrent mTBI and healthy controls.

As the precuneus is widely interconnected with frontal regions and is involved in executive functions and working memory, its role in cognition is crucial [69].

Furthermore, an interesting and significant age by group interaction within the right hippocampus was revealed in the present study, indicating an accelerated hippocampal volume loss in older participants due to recurrent traumatic events. This finding is in line with a study of Monti and colleagues (2013) in which a marginal age by group interaction within the right hippocampus in young to middle-aged subjects was found. As the hippocampus plays a key role in memory function, and volume loss within this brain region has been reported in association with memory loss in various pathological conditions such as depressions [70], mild cognitive impairment (MCI), and AD [71], the cognitive deficits in older participants with recurrent mTBI may be attributable to the observed changes in the hippocampal volume as well.

### 4.3. Alterations in FA and MD Values in Participants with mTBI

Evidence for the predominant myelin damage and axonal injury in the acute phase after TBI stems from animal studies, indicating a high vulnerability of white matter tracts such as the CC, which consists of long axons, to acceleration–deceleration forces [72,73]. Similar white matter alterations were noted in participants in the chronic phase after moderate-to-severe TBI [74,75]. Moreover, both whole-brain and regional differences between participants in the chronic phase after mTBI and healthy controls have been revealed [28,61,76]. In the sub-acute phase, evidence for increased FA in the bilateral superior frontal cortex has been found [25] using DTI as an advanced imaging technique for structural alterations.

However, several studies could not confirm the differences in healthy controls in FA [36,77] and MD [25,77] in the chronic phase after mTBI.

In line with Zhang and colleagues (2010) and Maruta and colleagues (2016), the present study revealed no whole-brain or regional differences in FA values between participants with recurrent mTBI in the chronic phase and healthy controls. Thus, even though the differences in the controls in the acute and sub-acute phase after mTBI seem to be a consistent finding, it is not yet clear if these changes persist into the chronic phase. Of note, differential results between studies might also be due to the impact of sample characteristics such as age, sports practised by participants, number of mTBI, injury, and symptom severity and type of injury as well as time since the last mTBI.

### 4.4. Functional Connectivity in Participants with mTBI

RSFC has been discussed as a possible biomarker for altered functional connectivity and plasticity due to recovery processes after brain injuries [40,41,78].

Based on a rat model for focal TBI in the primary motor cortex it has been shown that recovery in the acute phase is associated with a redistribution of functional representations in the brain [79]. Similarly, there is evidence for time- and recovery-dependent changes and functional reorganization in the human brain as well [38,40]. Studies reported hyper-connectivity in frontoparietal [38] and DMN networks [40] and decreased functional connectivity within the motor-striatal network [38] in the acute and sub-acute phase after mTBI. These changes have been discussed as being indicative of compensatory processes, which ultimately led to a normalization in the chronic phase (>6 months) [40]. In line with these results, Wang and colleagues (2011) found an improvement over time in most of the functional networks except the fornix. The results of the present study also support the hypothesis of normalization in the chronic phase by showing no significant difference between groups and no significant age by group interaction.

### 4.5. Limitations and Strengths

When interpreting the present results, several limitations have to be taken into account:

First, by relying on self-reports concerning the number of mTBI and symptoms, we might have over- or underestimated mTBI. However, standardized and validated questionnaires were used, which is the current standard procedure in mTBI research [20,76]. Besides, studies with healthy older adults, participants with MCI [80], and former athletes [81] could confirm the high reliability of self-reported TBI over time.

Second, one of the main inclusion criteria for participants with mTBI comprised the occurrence of at least two mTBI without generally limiting the number, which consequently could have led to a rather heterogeneous sample (range of mTBI: 2–7). However, as the aim of the present study was to examine the impact of recurrent mTBI, we defined our inclusion criteria in accordance with previously conducted studies addressing similar questions and thus, included participants with similar numbers of mTBI in our study [11,13,76].

Third, the healthy control group was recruited in the same local sports clubs as the mTBI group in order to minimize the baseline differences between groups. Hence, sub-concussive events may have led to alterations in the white matter integrity [82] and RSFC [83] and, therefore, diluted the differences between groups.

Fourth, more sensitive standardized computerized tests for selective and divided attention could have been conducted in order to obtain group differences. Please note that the negative results on this ‘standard battery of cognitive tests’, as successfully used in lifestyle studies in order to detect changes in cognition [84,85], render any detrimental effects of recurrent mTBI on everyday life functions highly unlikely.

Fifth, the older cohort is skewed towards female participants (young cohort: 4 female participants versus older cohort: 16 female participants). Current research indicates that females perform better in verbal cognitive tests than males [86]. However, as we found differences in verbal cognitive tests between healthy controls and participants with mTBI only for the RWT, but not for other verbal tests including the AVLT, it supports previous findings of verbal fluency being a sensitive measure for detecting cognitive deficits after recurrent mTBI [11].

Strengths of the present study include the multimodal approach, using different methods to investigate the impact of recurrent mTBI. Also, as far as we know, this study is the first to address the interaction between age and recurrent mTBI.

## 5. Conclusions

The present multimodal assessment did not reveal extensive differences between participants in the chronic phase after recurrent mTBI and group-wise matched healthy controls. Most notably, it did not reveal an aggravation of age-related processes in the older cohort of participants due to recurrent mTBI, as had been suggested in the literature [13,76,87]. However, our study was not designed to answer the question whether recurrent mTBI increased the incidence of dementia in older age, an issue of great relevance for healthcare systems that should be addressed in future studies with long-term follow-ups.

## Figures and Tables

**Table 1:** The young patients’ characteristics and older patients’ characteristics.

Young Patients’ Characteristics						Older Patients’ Characteristics					
**Sex**	**Age**	Years of Education	No. of mTBI	Time since Last mTBI (month)	Cause of Last mTBI	Sex	Age	Years of Education	No. of mTBI	Time since Last mTBI (month)	Cause of Last mTBI
m	27	17	2	N/A	Ice hockey	f	58	13	2	440	Bicycle accident
m	29	18	2	13	Ice hockey	m	69	18	4	106	Bicycle accident
f	22	15.5	4	27	Skating	f	55	13	2	366	Ski accident
m	21	15.5	3	33	Rugby	f	67	15.5	2	646	Accident
f	26	17.5	5	35-36	Fall	m	54	16	2	410	Accident
m	26	15.5	3	54	Football	m	80	13	3	580	Vehicle accident
m	24	17.5	2	27–30	Football	f	57	13	3	433	Accident
m	20	15.5	2	14	Fall	m	63	20	4	436	Boxing
m	21	15	2	9	Soccer	f	70	14	2	531	Bicycle accident
m	23	15	3	10	Football	m	57	18	3	62	Accident
m	21	15.5	2	22	Football	f	55	16	2	244	Accident
m	25	13	2	35–36	Football	m	55	18	2	204	Handball
m	23	10	2	12	Football	f	62	13	2	462	Accident
m	24	15.5	3	27–30	Football	f	59	17.5	2	489	Assault
m	25	13	6	7	Football	m	76	16	3	668	Boxing
m	30	18	7	11	Mountainbike	m	65	17.5	2	484	Soccer
m	24	15.5	2	32–44	Kickboxing	m	63	14.5	2	712	Accident

Note: m = male, f = female, mTBI = mild traumatic brain injury.

**Table 2:** The demographic data and results of the post hoc Student’s *t*-tests for verbal fluency.

	Young (*N* = 38)				Old (*N* = 33)				All (*N* = 71)			
	mTBI	Healthy	*T*	*p*	mTBI	Healthy	*T*	*p*	mTBI	Healthy	*T*	*p*
	Demographic data											
Sex	2 female, 15 male	2 female, 19 male	-	-	8 female, 9 male	8 female, 8 male	-	-	10 female, 24 male	10 female, 27 male	-	-
Age	24.2 ± 2.8	25.8 ± 5.4	−1.9	0.3	62.7 ± 7.7	61.7 ± 5.9	0.4	0.7	43.4 ± 3.5	41.3 ± 3.1	0.5	0.7
Education	15.4 ± 2.0	15.1 ± 1.7	0.5	0.6	15.7 ± 2.3	15.5 ± 1.9	0.2	0.8	15.4 ± 2.1	15.3 ± 1.8	0.5	0.6
MMSE	29.8 ± 0.4	30.0 ± 0.0	−1.9	0.1	29.5 ± 1.0	29.2 ± 1.4	0.8	0.4	29.7 ± 0.8	29.6 ± 1.0	0.1	0.7
BDI	2.5 ± 3.2	2.2 ± 2.4	0.3	0.8	2.7 ± 2.5	2.6 ± 2.3	0.2	0.9	2.6 ± 2.9	2.4 ± 2.3	0.4	0.7
	Verbal fluency											
RWT, Verbal fluency (s; food)	−0.95 ± 1.09	0 ± 1	−2.68	0.01	−0.12 ± 0.84	0 ± 1	−0.37	0.72	−0.42 ± 0.81	0 ± 1	−1.88	0.06
RWT, Verbal flexibility (gr; clothes/flowers)	−0.57 ± 0.87	0 ± 1	−1.77	0.08	−0.05 ± 1.31	0 ± 1	−0.13	0.90	−0.34 ± 0.94	0 ± 1	−1.43	0.16
RWT, overall Verbal fluency score	−0.99 ± 1.08	0 ± 1	−2.79	0.01	−0.08 ± 0.93	0 ± 1	−0.23	0.82	−0.43 ± 0.83	0 ± 1	−1.92	0.06

Note: Cognitive test scores reflect mean Z-scores. Groups were compared using Student’s *t*-test for independent samples [uncorrected *p*-values]. mTBI = mild traumatic brain injury. MMSE = Mini Mental Status Examination, BDI = Beck Depression Inventory, RWT = Regensburg Verbal Fluency Test.

**Table 3:** The results of the 2 × 2 MANOVA for ROI-specific FA values with the group (participants with mTBI versus healthy controls) and age (young versus old) as between-subject factors.

Independent Variables	Dependent Variables	df	F	*p*	η^2^
Group	FA fornix	1	1.12	0.29	0.02
	FA lUF	1	0.05	0.82	0.00
	FA rUF	1	0.04	0.85	0.00
	FA CC	1	0.18	0.68	0.00
Age	FA fornix	1	36.67	0.00	0.37
	FA lUF	1	4.25	0.04	0.06
	FA rUF	1	2.09	0.15	0.03
	FA CC	1	30.64	0.00	0.33
Age*Group	FA fornix	1	0.32	0.58	0.01
	FA lUF	1	0.98	0.33	0.02
	FA rUF	1	0.18	0.67	0.00
	FA CC	1	0.01	0.91	0.00

Note. MANOVA = multivariate analysis of variance, ROI = regions of interests, FA = fractional anisotropy, mTBI = mild traumatic brain injury, l/rC = left/right cingulum, l/rUF = left/right uncinate fasciculus, l/rCST = left/right corticospinal tract, CC = corpus callosum, l/rC = left/right cingulum, df = degrees of freedom, η^2^ = partial eta squared, Age*Group = age by group interaction.

**Table 4:** The results of the 2 × 2 MANOVA for ROI-specific mean diffusivity (MD) values with the group (participants with mTBI versus healthy controls) and age (young versus old) as between-subject factors.

Independent Variables	Dependent Variables	df	F	*p*	η^2^
Group	MD fornix	1	0.17	0.68	0.00
	MD lUF	1	0.00	1.00	0.00
	MD rUF	1	1.10	0.30	0.02
	MD CC	1	0.19	0.67	0.00
Age	MD fornix	1	25.34	0.00	0.29
	MD lUF	1	21.98	0.00	0.26
	MD rUF	1	12.79	0.00	0.17
	MD CC	1	9.14	0.00	0.13
Age*Group	MD fornix	1	1.10	0.30	0.02
	MD lUF	1	0.00	0.97	0.00
	MD rUF	1	0.00	0.98	0.00
	MD CC	1	0.68	0.41	0.01

Note. MANOVA = multivariate analysis of variance, ROI = regions of interests, mTBI = mild traumatic brain injury, MD = mean diffusivity, l/rUF = left/right uncinate fasciculus, CC = corpus callosum, df = degrees of freedom, η^2^ = partial eta squared, Age*Group = age by group interaction.

## Supplementary Materials

The following are available online at http://www.mdpi.com/2077-0383/7/5/95/s1, Supplementary Table 2 Results of the whole-brain voxel-based analysis comparing local gray matter volume between the different groups split with regards to: (A) Group (participants with mTBI versus healthy controls), (B) Age (young versus older subjects), and (C) interaction between age and group; Supplementary Table 3 Results of the whole-brain analysis comparing RSFC of the (A) right and (B) left medial OFC, the (C) right and (D) left precuneus, the (E) right and (F) left putamen, and the (G) right and (H) left hippocampus between the different groups split with regards to: group (participants with mTBI versus healthy controls), age (young versus older subjects), and interaction between age and group (participants with mTBI young > older > healthy controls young > older).

## References

[1] Smith S. Did Concussions Play Role in Lou Gehrig’s Disease?. http://edition.cnn.com/2010/HEALTH/08/17/als.lou.gehrigs.concussions/.

[2] Smith S. Ex-NFL Stars after Concussion: Lives Unraveled. http://edition.cnn.com/2010/HEALTH/11/24/fred.mcneill.concussions/.

[3] McCrea M., Guskiewicz K., Doncevic S., Helmick K., Kennedy J., Boyd C., Asmussen S., Ahn K.W., Wang Y., Hoelzle J. (2014). Day of injury cognitive performance on the Military Acute Concussion Evaluation (MACE) by U.S. military service members in OEF/OIF. Mil. Med..

[4] King N.S., Crawford S., Wenden F.J., Wade D.T. (1995). The Rivermead Post Concussion Symptoms Questionnaire: A measure of symptoms commonly experienced after head injury and its reliability. J. Neurol..

[5] Kinnunen K.M., Greenwood R., Powell J.H., Leech R., Hawkins P.C., Bonnelle V., Patel M.C., Counsell S.J., Sharp D.J. (2010). White matter damage and cognitive impairment after traumatic brain injury. Brain.

[6] Konrad C., Geburek A.J., Rist F., Blumenroth H., Fischer B., Husstedt I., Arolt V., Schiffbauer H., Lohmann H. (2011). Long-term cognitive and emotional consequences of mild traumatic brain injury. Psychol. Med..

[7] Vanderploeg R.D., Curtis G., Belanger H.G. (2005). Long-term neuropsychological outcomes following mild traumatic brain injury. J. Int. Neuropsychol. Soc..

[8] Wilke S., List J., Mekle R., Lindenberg R., Bukowski M., Ott S., Schubert F., Ittermann B., Flöel A. (2016). No effect of anodal transcranial direct current stimulation on gamma-aminobutyric acid levels in patients with recurrent mild traumatic brain injury. J. Neurotrauma.

[9] List J., Ott S., Bukowski M., Lindenberg R., Flöel A. (2015). Cognitive function and brain structure after recurrent mild traumatic brain injuries in young-to-middle-aged adults. Front. Hum. Neurosci..

[10] Keightley M.L., Saluja R.S., Chen J.K., Gagnon I., Leonard G., Petrides M., Ptito A. (2014). A functional magnetic resonance imaging study of working memory in youth after sports-related concussion: Is it still working?. J. Neurotrauma.

[11] Tremblay S., De Beaumont L., Henry L.C., Boulanger Y., Evans A.C., Bourgouin P., Poirier J. (2013). Théoret, H.; Lassonde, M.; Sports concussions and aging: A neuroimaging investigation. Cereb. Cortex.

[12] Gomez R.G., White D.A. (2006). Using verbal fluency to detect very mild dementia of the Alzheimer type. Arch. Clin. Neuropsychol..

[13] De Beaumont L., Théoret H., Mongeon D., Messier J., Leclerc S., Tremblay S., Ellemberg D., Lassonde M. (2009). Brain function decline in healthy retired athletes who sustained their last sports concussion in early adulthood. Brain.

[14] Moretti L., Cristofori I., Weaver S.M., Chau A., Portelli J.N., Grafman J. (2012). Cognitive decline in older adults with a history of traumatic brain injury. Lancet Neurol..

[15] Plassman B.L., Havlik R.J., Steffens D.C., Helms M.J., Newman T.N., Drosdick D., Phillips C., Gau B.A., Welsh-Bohmer K.A., Burke J.R. (2000). Documented head injury in early adulthood and risk of Alzheimer’s disease and other dementias. Neurology.

[16] Shively S., Scher A.I., Perl D.P., Diaz-Arrastia R. (2012). Dementia resulting from traumatic brain injury. Arch. Neurol..

[17] Carroll L.J., Cassidy J.D., Holm L., Kraus J., Coronado V.G. (2004). Methodological issues and research recommendations for mild traumatic brain injury: The WHO Collaborating Centre Task Force on Mild Traumatic Brain Injury. J. Rehabil. Med. Med..

[18] Keightley M.L., Sinopoli K.J., Davis K.D., Mikulis D.J., Wennberg R., Tartaglia M.C., Chen J.K., Tator C.H. (2014). Is there evidence for neurodegenerative change following traumatic brain injury in children and youth? A scoping review. Front. Hum. Neurosci..

[19] Wilde E.A., Merkley T.L., Bigler E.D., Max J.E., Schmidt A.T., Ayoub K.W., McCauley S.R., Hunter J.V., Hanten G., Li X. (2012). Longitudinal changes in cortical thickness in children after traumatic brain injury and their relation to behavioral regulation and emotional control. Int. J. Dev. Neurosci..

[20] Monti J.M., Voss M.W., Pence A., McAuley E., Kramer A.F., Cohen N.J. (2013). History of mild traumatic brain injury is associated with deficits in relational memory, reduced hippocampal volume, and less neural activity later in life. Front. Aging Neurosci..

[21] Brezova V., Moen K.G., Skandsen T., Vik A., Brewer J.B., Salvesen Ø., Håberg A.K. (2014). Prospective longitudinal MRI study of brain volumes and diffusion changes during the first year after moderate to severe traumatic brain injury. NeuroImage Clin..

[22] Lannsjö M., Raininko R., Bustamante M., von Seth C., Borg J. (2013). Brain pathology after mild traumatic brain injury: An exploratory study by repeated magnetic resonance examination. J. Rehabil. Med..

[23] Slemmer J.E. (2002). Repeated mild injury causes cumulative damage to hippocampal cells. Brain.

[24] King J.B., Lopez-Larson M.P., Yurgelun-Todd D.A. (2016). Mean cortical curvature reflects cytoarchitecture restructuring in mild traumatic brain injury. NeuroImage Clin..

[25] Ling J.M., Klimaj S., Toulouse T., Mayer A.R. (2013). A prospective study of gray matter abnormalities in mild traumatic brain injury. Neurology.

[26] Rutgers D.R., Toulgoat F., Cazejust J., Fillard P., Lasjaunias P., Ducreux D. (2008). White matter abnormalities in mild traumatic brain injury: A diffusion tensor imaging study. Am. J. Neuroradiol..

[27] Gale S.D., Johnson S.C., Bigler E.D., Blatter D.D. (1995). Trauma-induced degenerative changes in brain injury: A morphometric analysis of three patients with preinjury and postinjury MR scans. J. Neurotrauma.

[28] Henry L.C., Tremblay J., Tremblay S., Lee A., Brun C., Lepore N., Theoret H., Ellemberg D., Lassonde M. (2011). Acute and chronic changes in diffusivity measures after sports concussion. J. Neurotrauma.

[29] Seo J.P., Jang S.H. (2015). Traumatic axonal injury of the corticospinal tract in the subcortical white matter in patients with mild traumatic brain injury. Brain Inj..

[30] Kraus M.F., Susmaras T., Caughlin B.P., Walker C.J., Sweeney J.A., Little D.M. (2007). White matter integrity and cognition in chronic traumatic brain injury: A diffusion tensor imaging study. Brain.

[31] Inglese M., Makani S., Johnson G., Cohen B.A., Silver J.A., Gonen O., Grossman R.I. (2005). Diffuse axonal injury in mild traumatic brain injury: A diffusion tensor imaging study. J. Neurosurg..

[32] Lipton M.L., Gellella E., Lo C., Gold T., Ardekani B.A., Shifteh K., Bello J.A., Branch C.A. (2008). Multifocal white matter ultrastructural abnormalities in mild traumatic brain injury with cognitive disability: A voxel-wise analysis of diffusion tensor imaging. J. Neurotrauma.

[33] Lo C., Shifteh K., Gold T., Bello J.A., Lipton M.L. (2009). Diffusion tensor imaging abnormalities in patients with mild traumatic brain injury and neurocognitive impairment. J. Comput. Assist. Tomogr..

[34] Geary E.K., Kraus M.F., Pliskin N.H., Little D.M. (2010). Verbal learning differences in chronic mild traumatic brain injury. J. Int. Neuropsychol. Soc..

[35] Niogi S.N., Mukherjee P., Ghajar J., Johnson C., Kolster R.A., Sarkar R., Lee H., Meeker M., Zimmerman R.D., Manley G.T. (2008). Extent of microstructural white matter injury in postconcussive syndrome correlates with impaired cognitive reaction time: A 3T diffusion tensor imaging study of mild traumatic brain injury. Am. J. Neuroradiol..

[36] Zhang K., Johnson B., Pennell D., Ray W., Sebastianelli W., Slobounov S. (2010). Are functional deficits in concussed individuals consistent with white matter structural alterations: Combined FMRI & DTI study. Exp. Brain Res..

[37] Palacios E.M., Sala-Llonch R., Junque C., Roig T., Tormos J.M., Bargallo N., Vendrell P. (2013). Resting-state functional magnetic resonance imaging activity and connectivity and cognitive outcome in traumatic brain injury. JAMA Neurol..

[38] Shumskaya E., Teuntje M.J.C., Norris D.G., Vos P.E. (2012). Abnormal whole-brain functional networks in homogeneous acute mild traumatic brain injury. Neurology.

[39] Zhou Y., Milham M.P., Lui Y.W., Miles L., Reaume J., Sodickson D.K., Grossman R.I., Ge Y. (2012). Default-mode network disruption in mild traumatic brain injury. Radiology.

[40] Bharath R.D., Munivenkatappa A., Gohel S., Panda R., Saini J., Rajeswaran J., Shukla D., Bhagavatula I.D., Biswal B.B. (2015). Recovery of resting brain connectivity ensuing mild traumatic brain injury. Front. Hum. Neurosci..

[41] Zhu D.C., Covassin T., Nogle S., Doyle S., Russell D., Pearson R.L., Monroe J., Liszewski C.M., DeMarco K.J., Kaufman D.I. (2015). A potential biomarker in sports-related concussion: Brain functional connectivity alteration of the default-mode network measured with sequential resting-state fMRI. J. Neurotrauma.

[42] Konstantinou N., Pettemeridou E., Seimenis I., Eracleous E., Papacostas S.S., Papanicolaou A.C., Constantinidou F. (2016). Assessing the relationship between neurocognitive performance and brain volume in chronic moderate-severe traumatic brain injury. Front. Neurol..

[43] Folstein M., Folstein S., McHugh P. (1975). “Mini-mental state”. A practical method for grading the cognitive state of patients for the clinician. J. Psychiatr. Res..

[44] Hautzinger M., Keller F., Kühner C. (2006). Beck Depression Inventar II (BDI 2).

[45] Vanderploeg R.D., Curtiss G., Luis C.A., Salazar A.M. (2007). Long-term morbidities following self-reported mild traumatic brain injury. J. Clin. Exp. Neuropsychol..

[46] Matser E.J.T., Lezak M.D., Jordan B.D., Traumatic H., In B. (1999). Neuropsychological impairment in amateur soccer players. JAMA.

[47] Belanger H.G., Vanderploeg R.D. (2005). The neuropsychological impact of sports-related concussion: A meta-analysis. J. Int. Neuropsychol. Soc..

[48] Helmstaedter C., Lendt M., Lux S. (2001). Verbaler Lern- und Merkfähigkeitstest (VLMT).

[49] Reitan R.M. (1958). Validity of the Trail Making Test as an indicator or organic brain damage. Percept. Mot. Skills.

[50] Aschenbrenner S., Tucha O., Lange K. (2000). Regensburger Wortflüssigkeits-Test, Handanweisung [Manual for the Regensburger Word Fluency Test].

[51] Osterrieth P.A. (1944). Le test de copie d’une figure complex: Contribution a l’etude de la perception et de la memoire. Arch. Psychol..

[52] Härting C., Markowitsch H., Neufeld H., Calabrese P., Deisinger K., Kessler J. (2000). Wechsler Gedächtnistest—Revidierte Fassung WMS-R, Deutsche Adaptation (Manual for the Wechsler Memory Scale—Revised, German Adaptation).

[53] Tzourio-Mazoyer N., Landeau B., Papathanassiou D., Crivello F., Etard O., Delcroix N., Mazoyer B., Joliot M. (2002). Automated anatomical labeling of activations in SPM using a macroscopic anatomical parcellation of the MNI MRI single-subject brain. Neuroimage.

[54] Behrens T.E.J., Woolrich M.W., Jenkinson M., Johansen-Berg H., Nunes R.G., Clare S., Matthews P.M., Brady J.M., Smith S.M. (2003). Characterization and propagation of uncertainty in diffusion-weighted MR imaging. Magn. Reson. Med..

[55] Smith S.M., Jenkinson M., Johansen-Berg H., Rueckert D., Nichols T.E., Mackay C.E., Watkins K.E., Ciccarelli O., Cader M.Z., Matthews P.M. (2006). Tract-based spatial statistics: Voxelwise analysis of multi-subject diffusion data. Neuroimage.

[56] Antonenko D., Külzow N., Cesarz M.E., Schindler K., Grittner U., Flöel A. (2016). Hippocampal pathway plasticity is associated with the ability to form novel memories in older adults. Front. Aging Neurosci..

[57] Panenka W.J., Lange R.T., Bouix S., Shewchuk J.R., Manraj K., Heran S., Brubacher J.R., Eckbo R., Shenton M.E., Iverson G.L. (2015). Neuropsychological outcome and diffusion tensor imaging in complicated versus uncomplicated mild traumatic brain injury. PLoS ONE.

[58] Yan C.-G., Zhang Y.-F. (2010). DPARSF: A MATLAB toolbox for “pipeline” data analysis of resting-state fMRI. Front. Syst. Neurosci..

[59] Guskiewicz K.M., Ross S.E., Marshall S.W. (2001). Postural stability and neuropsychological deficits after concussion in collegiate athletes. J. Athl. Train..

[60] McCauley S.R., Wilde E.A., Barnes A., Hanten G., Hunter J.V., Levin H.S., Smith D.H. (2014). Patterns of early emotional and neuropsychological sequelae after mild traumatic brain injury. J. Neurotrauma.

[61] Wada T., Asano Y., Shinoda J. (2012). Decreased fractional anisotropy evaluated using tract-based spatial statistics and correlated with cognitive dysfunction in patients with mild traumatic brain injury in the chronic stage. Am. J. Neuroradiol..

[62] Mckee A.C., Daneshvar D.H. (2015). The neuropathology of traumatic brain injury. Handb. Clin. Neurol..

[63] Toledo E., Lebel A., Becerra L., Minster A., Linnman C., Maleki N., Dodick D.W., Borsook D. (2012). The young brain and concussion: Imaging as a biomarker for diagnosis and prognosis. Neurosci. Biobehav. Rev..

[64] MacKenzie J.D., Siddiqi F., Babb J.S., Bagley L.J., Mannon L.J., Sinson G.P., Grossman R.I. (2002). Brain atrophy in mild or moderate traumatic brain injury: A longitudinal quantitative analysis. AJNR Am. J. Neuroradiol..

[65] Palacios E.M., Sala-Llonch R., Junque C., Fernandez-Espejo D., Roig T., Tormos J.M., Bargallo N., Vendrell P. (2013). Long-term declarative memory deficits in diffuse TBI: Correlations with cortical thickness, white matter integrity and hippocampal volume. Cortex.

[66] Wang X., Xie H., Cotton A.S., Tamburrino M.B., Brickman K.R., Lewis T.J., Mclean S.A., Liberzon I. (2015). Early cortical thickness change after mild traumatic brain injury following motor vehicle collision. J. Neurotrauma.

[67] Wilde E.A., Newsome M.R., Bigler E.D., Pertab J., Merkley T.L., Hanten G., Scheibel R.S., Li X., Chu Z., Yallampalli R. (2011). Brain imaging correlates of verbal working memory in children following traumatic brain injury. Int. J. Psychophysiol..

[68] Hudak A., Warner M., Marquez de la Plata C., Moore C., Harper C., Diaz-Arrastia R. (2011). Brain morphometry changes and depressive symptoms after traumatic brain injury. Psychiatry Res. Neuroimaging.

[69] Vogt B.A., Laureys S. (2005). Posterior cingulate, precuneal & retrosplenial cortices: Cytology & components of the neural network correlates of consciousness. Prog. Brain Res..

[70] Hickie I., Naismith S., Ward P.B., Turner K., Scott E., Mitchell P., Wilhelm K., Parker G. (2005). Reduced hippocampal volumes and memory loss in patients with early- and late-onset depression. Br. J. Psychiatry.

[71] Schuff N., Woerner N., Boreta L., Kornfield T., Shaw L.M., Trojanowski J.Q., Thompson P.M., Jack C.R., Weiner M.W. (2009). MRI of hippocampal volume loss in early Alzheimer’s disease in relation to ApoE genotype and biomarkers. Brain.

[72] Armstrong R.C., Mierzwa A.J., Marion C.M., Sullivan G.M. (2016). White matter involvement after TBI: Clues to axon and myelin repair capacity. Exp. Neurol..

[73] Mierzwa A.J., Marion C.M., Sullivan G.M., McDaniel D.P., Armstrong R.C. (2015). Components of myelin damage and repair in the progression of white matter pathology after mild traumatic brain injury. J. Neuropathol. Exp. Neurol..

[74] Green R.E.A., Colella B., Maller J.J., Bayley M., Glazer J., Mikulis D.J. (2014). Scale and pattern of atrophy in the chronic stages of moderate-severe TBI. Front. Hum. Neurosci..

[75] Bendlin B.B., Ries M.L., Lazar M., Alexander A.L., Dempsey R.J., Rowley H.A., Sherman J.E., Johnson S.C. (2008). Longitudinal changes in patients with traumatic brain injury assessed with diffusion-tensor and volumetric imaging. Neuroimage.

[76] Tremblay S., Henry L.C., Bedetti C., Larson-Dupuis C., Gagnon J.-F., Evans A.C., Théoret H., Lassonde M., De Beaumont L. (2014). Diffuse white matter tract abnormalities in history of sports-related concussions. Brain J. Neurol..

[77] Maruta J., Palacios E.M., Zimmerman R.D., Ghajar J., Mukherjee P. (2016). Chronic post-concussion neurocognitive deficits. I. Relationship with white matter integrity. Front. Hum. Neurosci..

[78] Nudo R.J. (2013). Recovery after brain injury: Mechanisms and principles. Front. Hum. Neurosci..

[79] Nishibe M., Barbay S., Guggenmos D., Nudo R.J. (2010). Reorganization of motor cortex after controlled cortical impact in rats and implications for functional recovery. J. Neurotrauma.

[80] Wilmoth K., LoBue C., Clem M., Didehbani N., Hart J., Womack K., Bell K., Batjer H., Cullum C. (2016). Reliability of Self-Reported Concussion History in Older Adults with and Without Cognitive Impairment. Arch. Clin. Neuropsychol..

[81] Didehbani N., Wilmoth K., Fields L., Lobue C., Strain J., Spence J., Dieppa M., Cullum M., Hart J. (2017). Reliability of Self-Reported Concussion History in Retired NFL Players. Ann. Sport Med. Res..

[82] Koerte I.K., Ertl-Wagner B., Reiser M., Zafonte R., Shenton M.E. (2012). White matter integrity in the brains of professional soccer players without a symptomatic concussion. JAMA.

[83] Johnson B., Neuberger T., Gay M., Hallett M., Slobounov S. (2014). Effects of subconcussive head trauma on the default mode network of the brain. J. Neurotrauma.

[84] Witte A.V., Köbe T., Kerti L., Rujescu D., Flöel A. (2016). Impact of KIBRA Polymorphism on Memory Function and the Hippocampus in Older Adults. Neuropsychopharmacology.

[85] Köbe T., Witte A.V., Schnelle A., Tesky V.A., Pantel J., Schuchardt J.P., Hahn A., Bohlken J., Grittner U., Flöel A. (2017). Impact of resveratrol on glucose control, hippocampal structure and connectivity, and memory performance in patients with mild cognitive impairment. Front. Neurosci..

[86] Sundermann E.E., Maki P.M., Rubin L.H., Lipton R.B., Landau S., Biegon A. (2016). Female advantage in verbal memory: Evidence of sex-specific cognitive reserve. Neurology.

[87] Broglio S.P., Eckner J.T., Paulson H.L., Kutcher J.S. (2012). Cognitive decline and aging: The role of concussive and subconcussive impacts. Exerc. Sport Sci. Rev..

