# Supplementary material for: Multimodal Assessment of Recurrent MTBI across the Lifespan
Source: J Clin Med. 2018 May 1;7(5):95. doi: 10.3390/jcm7050095 (PMC5977134; doi:10.3390/jcm7050095)
Supplement: Supplementary file 1 [file jcm-07-00095-s001.pdf]

Supplementary Material

1. Experimental Section

1.2 Magnetic Resonance Imaging

1.2.1 Voxel-based morphometry

Data preprocessing consisted of tissue classification and segmentation into gray and white matter, image registration, as well as bias correction for magnetic field inhomogeneities. Additionally, Hidden Markov Random Fields (HMRF) were applied to increase the signal-to-noise ratio of the final tissue maps. HMRF provide spatial constraints on tissue segmentation based on the intensities of neighboring voxels. Specifically voxels which are isolated and unlikely to be associated with a certain tissue class are removed from the final tissue maps [1]. All resulting gray and white matter images were registered to a template provided by the International Consortium of Brain Mapping, and a diffeomorphic image registration algorithm (DARTEL) [2] was used for spatially normalizing tissue maps into stereotactic Montreal Neurological Institute (MNI) space. Finally, normalized gray matter maps (m0wrp1*), depicting the absolute amount of regional gray matter (GM) volume corrected for individual brain sizes, were smoothed with a standard 8 mm full-width-at-half-maximum (FWHM) [3] isotropic Gaussian kernel and used for further statistical analyses.

1.2.2 Tract-based spatial statistics and probabilistic tractography

Several processing steps were conducted: 1) Nonlinear alignment of all subjects' FA data into the FMRIB58_FA standard space, 2) Affine-transformation of these aligned images into MNI152 standard space (1 x 1 x 1 mm), 3) Creating a 4D image by merging all of these subjects' FA images, 4) Skeletonisation of mean FA images and creating a mean FA skeleton and 5) Re-alignment of subjects' FA data onto this skeleton and voxel-wise cross-subject comparison using "Randomise" with threshold-free cluster enhancement and 5000 permutations.

1.2.3 Analysis of RSFC data

During preprocessing, images were first corrected for acquisition time difference between the slices, and then realigned to the first volume to correct for head motion between volumes. Physiological noise was reduced by regressing out signals from white matter, cerebrospinal fluid and the six head movement parameters and by removing a linear tread and band-pass filtering the data to (0.01–0.08 Hz) to reduce the effects of very low and high frequency physiological noise.

2. Results

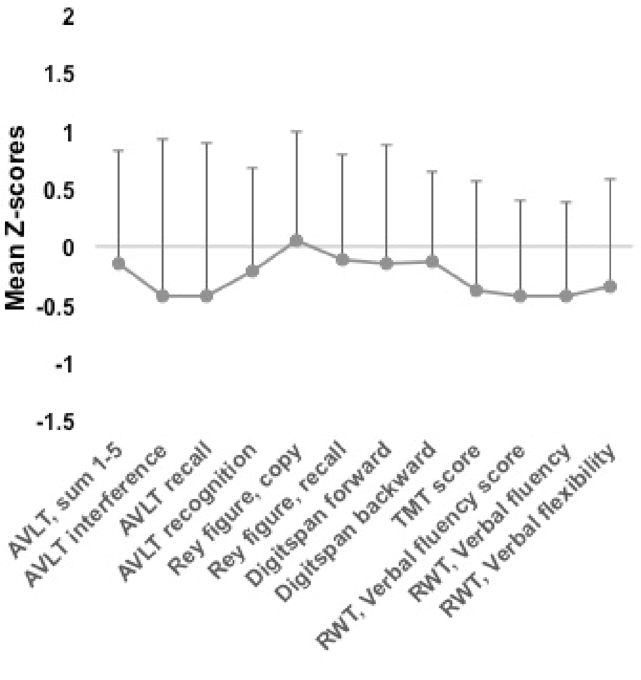

Figure 1. Cognitive profiles of all (young + older) participants with recurrent mTBI. Mean Z-scores (+1 standard deviation) of cognitive tests for participants with mTBI. mTBI = mild traumatic brain injury, AVLT = Auditory Verbal Learning Test; TMT-score = Trail Making Test score; RWT = Regensburg Verbal Fluency Test.

Table 1. Results of the post hoc Student's t-tests for the cognitive testing.

	Young (N=38)				Old (N=33)				All (N=71)			
	**mTBI**	**Healthy**	***T***	***p***	**mTBI**	**Healthy**	***T***	***p***	**mTBI**	**Healthy**	***T***	***p***
Verbal memory												
AVLT, sum 1-5	0.26 ± 0.71	0 ± 1	0.87	0.39	-0.50 ± 1.05	0 ± 1	-1.40	0.17	-0.15 ± 0.98	0 ± 1	-0.61	0.55
AVLT, 6-5	-0.38 ± 1.76	0 ± 1	-0.80	0.43	-0.50 ± 1.18	0 ± 1	-1.30	0.20	-0.44 ± 1.38	0 ± 1	-1.48	0.14
AVLT, 7-5 (delayed recall)	-0.31 ± 0.93	0 ± 1	-0.95	0.35	-0.60 ± 1.89	0 ± 1	-1.12	0.27	-0.42 ± 1.34	0 ± 1	-1.47	0.15
AVLT, Recognition	-0.29 ± 1.06	0 ± 1	-0.77	0.45	-0.20 ± 0.79	0 ± 1	-0.61	0.55	-0.21 ± 0.90	0 ± 1	-0.86	0.39
Visuospatial skills												
Rey figure, copy	0.44 ± 0.15	0 ± 1	1.79	0.08	-0.35 ± 1.21	0 ± 1	-0.89	0.38	0.05 ± 0.92	0 ± 1	0.25	0.81
Rey figure, recall	-0.14 ± 1.25	0 ± 1	-0.36	0.73	-0.08 ± 0.75	0 ± 1	-0.26	0.79	-0.11 ± 0.92	0 ± 1	-0.48	0.63
Processing speed												
TMT-A/B score	0.36 ± 1.11	0 ± 1	0.72	0.32	0.37 ± 0.73	0 ± 1	1.17	0.25	0.37 ± 9.5	0 ± 1	1.56	0.12
Working memory												
Digit Span, forward	0.13 ± 1.03	0 ± 1	0.39	0.70	-0.40 ± 0.81	0 ± 1	-1.24	0.22	-0.14 ± 1.04	0 ± 1	-0.58	0.56
Digit Span, backward	-0.05 ± 0.83	0 ± 1	-0.17	0.87	-0.18 ± 0.69	0 ± 1	-0.62	0.54	-0.14 ± 0.79	0 ± 1	-0.63	0.53

Note. Cognitive test scores reflect mean Z-scores. Groups were compared using Student's t-test for independent samples [uncorrected p-values]. mTBI= mild traumatic brain injury. MMSE = Mini Mental Status Examination, BDI = Beck Depression Inventory, TMT-A/B score = Trail Making Test, AVLT = Auditory Verbal Learning Test.

Supplementary Table 2| Results of the whole-brain voxel-based analysis comparing local gray matter volume between the different groups split with regards to: A) Group (participants with mTBI vs. healthy controls), B) Age (young vs. older subjects), and C) interaction between age and group.

**Anatomical region**	**L/R**	**Number of voxels in cluster**	**Z score of local maximum**	**MNI peak voxel coordinates**		
				**x**	**y**	**z**
**A) Group**						
Healthy controls > participants						
with mTBI	R	30	3.59	16	-64	40
Precuneus[a]	L	21	3.53	-14	64	-5
Medial OFC[a]						
Participants with mTBI > healthy controls		No suprathreshold clusters				
**B) Age**						
Young > older subjects						
Cerebellum	L	1050690	Inf	-4	-60	-8
Inferior parietal lobe	L	1290	6.43	-45	-37	48
Precentral gyrus	L	42	6.27	-33	-27	54
Supplementary motor cortex	R	63	6.03	3	-18	63
Postcentral gyrus	L	17	5.91	-28	-36	57
Precentral gyrus	R	16	5.86	34	-24	51
Supplementary motor cortex	L	3	5.51	-3	-15	61
Postcentral gyrus	R	3	4.77	24	-39	64
Hippocampus	L	68	4.70	-16	-15	-20
Superior parietal lobe	L	3	4.64	-20	-43	64
No ROI/Temporal pole	L	47	4.63	-40	11	-44
Superior parietal lobe	L	13	4.56	-21	-69	51
Superior parietal lobe	L	1	4.56	-34	-64	49
Superior parietal lobe	L	2	4.51	-34	-67	48

No ROI/Temporal pole	L	4	4.50	-45	2	-45
Superior occipital lobe	R	1	4.46	27	-82	28
Superior occipital lobe	R	1	4.43	30	-84	27
Older > young subjects		No suprathreshold clusters				
**C) Group x Age interactions**						
Participants with mTBI $^{young > older}$ > healthy controls $^{young > eldely}$						
Hippocampus[a]	R	5	3.16	38	-18	-23
Participants with mTBI $^{older > young}$ > healthy control $^{older > young}$		No suprathreshold clusters				

Reported clusters survived a voxel-wise family-wise error (FWE) correction at $p < 0.05$.

L, left hemisphere; R, right hemisphere.

[a]Voxel-wise small-volume corrected (SVC).

Supplementary Table 3| Results of the whole-brain analysis comparing RSFC of the A) right and B) left medial OFC, the C) right and D) left precuneus, the E) right and F) left putamen, and the G) right and H) left hippocampus between the different groups split with regards to: group (patients with mTBI vs. healthy controls), age (young vs. older subjects), and interaction between age and group (mTBI patients $^{\text{young > older}}$ > healthy controls $^{\text{young > older}}$).

**Anatomical region**	**L/R**	**Number of voxels in cluster**	**Z score of local maximum**	**MNI peak voxel coordinates**		
				**x**	**y**	**z**
**A) Right medial OFC**						
Healthy controls > mTBI patients		No suprathreshold clusters				
mTBI patients > healthy controls		No suprathreshold clusters				
Young > older subjects						
Middle temporal gyrus	L	97	5.02	-60	-15	-18
Medial superior frontal gyrus/orbital	R	124	4.90	3	54	-9
part	L	159	4.63	0	-63	21
Precuneus	R	65	4.19	15	39	48
Dorsolateral superior frontal gyrus						
Older > younger subjects						
Inferior frontal gyrus	R	137	4.79	54	9	9
Supplementary motor cortex	L	139	4.62	0	3	45
Middle frontal gyrus	L	38	4.52	-24	-6	48
Insula	L	110	4.30	-39	0	12
Age x Group interaction		No suprathreshold clusters				
**B) Left medial OFC**						
Healthy controls > mTBI patients		No suprathreshold clusters				
mTBI patients > healthy controls						
Temporal pole	L	61	4.45	-30	6	39
Young > older subjects						
Medial superior frontal gyrus/orbital	L	63	4.46	0	54	-9

part	L	40	4.33	-30	-39	0
Hippocampus	L	58	4.18	-57	-15	-18
Middle temporal gyrus						
Older > young subjects						
Inferior frontal gyrus	R	70	4.90	54	9	18
Inferior frontal gyrus	L	74	4.31	51	6	9
Middle frontal gyrus	L	68	4.25	-24	3	54
Middle frontal gyrus	L	46	4.11	-45	33	30
Supplementary motor cortex	R	54	3.91	3	6	57
Age x Group interaction		No suprathreshold clusters				
**C) Right precuneus**						
Healthy controls > mTBI patients		No suprathreshold clusters				
mTBI patients > healthy controls		No suprathreshold clusters				
Young > older subjects						
Thalamus	R	76	4.81	12	-24	12
No ROI/Paracingulate gyrus	L	47	4.04	-9	-30	30
Older > young subjects						
Postcentral gyrus	L	40	4.50	-27	-36	69
Postcentral gyrus	R	45	4.14	18	-33	72
Supplementary motor cortex	R	43	3.87	12	-24	51
Age x Group interaction		No suprathreshold clusters				
**D) Left precuneus**						
Healthy controls > mTBI patients		No suprathreshold clusters				
mTBI patients > healthy controls		No suprathreshold clusters				
Young > older subjects						
No ROI/Thalamus	L	83	5.14	-15	-33	9
No ROI	L	100	4.59	9	-30	21
No ROI/Hippocampus	L	36	4.44	-39	-9	-21
Older > young subjects						
No ROI/Inferior frontal gyrus	L	53	3.83	-54	21	-6
Age x Group interaction		No suprathreshold clusters				

**E) Right putamen**						
Healthy controls > mTBI patients		No suprathreshold clusters				
mTBI patients > healthy controls		No suprathreshold clusters				
Young > older subjects		No suprathreshold clusters				
Older > young subjetcs						
Hippocampus	L	63	4.98	21	-6	-27
Age x Group interaction		No suprathreshold clusters				
**F) Left putamen**						
Healthy controls > mTBI patients		No suprathreshold clusters				
mTBI patients > healthy controls		No suprathreshold clusters				
Young > older subjects		No suprathreshold clusters				
Older > young subjects						
Hippocampus/Parahippocampal gyrus	R	77	4.93	21	-6	-27
Age x Group interaction		No suprathreshold clusters				
**G) Right hippocampus**						
Healthy controls > mTBI patients		No suprathreshold clusters				
mTBI patients > healthy controls		No suprathreshold clusters				
Young > older subjects		No suprathreshold clusters				
Older > young subjects						
Inferior temporal gyrus	L	55	4.35	-39	-9	-42
Temporal pole	R	46	4.18	27	9	-39
Age x Group interaction		No suprathreshold clusters				
**H) Left hippocampus**						
Healthy controls > mTBI patients		No suprathreshold clusters				
mTBI patients > healthy controls		No suprathreshold clusters				
Young > older subjects						
Medial superior frontal gyrus/orbital	L	105	4.20	-9	39	-12
part	L	44	3.88	-15	-33	63
Medial frontal lobe/paracentral lobule	L	53	3.79	-24	39	51

No ROI/Dorsolateral superior frontal gyrus	
Older > young subjects	No suprathreshold clusters
Age x Group interaction	No suprathreshold clusters

Reported clusters survived a voxel-wise family-wise error (FWE) correction using an uncorrected cluster-defining threshold of $p < 0.001$.

L, left hemisphere; R, right hemisphere.

3. References

1. Zhang, Y.; Brady, M.; Smith, S. Segmentation of brain MR images through a hidden Markov random field model and the expectation-maximization algorithm. IEEE Trans. Med. Imaging 2001, 20, 45–57, doi:10.1109/42.906424.
2. Ashburner, J. A fast diffeomorphic image registration algorithm. Neuroimage 2007, 38, 95–113, doi:10.1016/j.neuroimage.2007.07.007.
3. Silver, M.; Montana, G.; Nichols, T.E. False positives in neuroimaging genetics using voxel-based morphometry data. Neuroimage 2011, 54, 992–1000, doi:10.1016/j.neuroimage.2010.08.049.
